# Meta-Research: Broadening the Scope of *PLOS Biology*

**DOI:** 10.1371/journal.pbio.1002334

**Published:** 2016-01-04

**Authors:** Stavroula Kousta, Christine Ferguson, Emma Ganley

**Affiliations:** *PLOS Biology*, Public Library of Science, Cambridge, United Kingdom

## Abstract

In growing recognition of the importance of how scientific research is designed, performed, communicated, and evaluated, *PLOS Biology* announces a broadening of its scope to cover meta-research articles.

The scientific examination of how research is designed, carried out, communicated, and evaluated determines how much confidence we can have in the insights that ultimately arise from it. This understanding underlies our decision to expand the scope of the research section of *PLOS Biology* to include meta-research articles.

It has become increasingly apparent that the failure to reproduce results is a significant problem across the biomedical sciences. In a seminal article, Ioannidis used simulations to demonstrate that, given current research practices, research claims are more likely to be false than true [1]. A recent effort to replicate 100 psychology studies found that only 39% could be replicated, with replication effects overall having half the magnitude of original effects [2]. Chalmers and Glasziou estimated that approximately 85% of research investment in the biomedical sciences–or US$200 billion of the worldwide investment in 2010 –is wasted [3]. Freedman and colleagues estimated that over 50% of preclinical research can’t be replicated, placing the approximate annual cost of irreproducibility in the US alone at US$28 billion [4]. Unsurprisingly, drug discovery has slowed and its costs have risen, as preclinical interventions in animal models are rarely recapitulated in clinical trials [5].

Trust in the scientific enterprise has been seriously undermined, and this has not been helped in recent years by numerous retractions (see Retraction Watch for reporting [6]). There is an urgent need to address this credibility crisis and improve the standards of research practices. The emerging field of meta-research aims to characterize existing standards and identify improved practices, in the hope of raising awareness and ultimately improving the quality and reliability of scientific research [7].

The new meta-research section of *PLOS Biology* will be data-driven and feature experimental, observational, modelling, and meta-analytic research that addresses issues related to the design, methods, reporting, verification, and evaluation of research. It will also encompass research into the systems that evaluate and reward individual scientists and institutions. We welcome both exploratory and confirmatory research that has the potential to drive change in research and evaluation practices in the life sciences and beyond. The themes include, but are not limited to, transparency, established and novel methodological standards, sources of bias (conflicts of interest, selection, inflation, funding, etc.), data sharing, evaluation metrics, assessment, reward, and funding structures.

To support our consideration of meta-research articles we have recently added several experts in this area to *PLOS Biology*’s Editorial Board. These include, but will not be limited to, Lisa Bero (University of Sydney); Isabelle Boutron (Université Paris Descartes); Ulrich Dirnagl (Charité—Universitätsmedizin Berlin); John PA Ioannidis (Stanford University); Jonathan Kimmelman (McGill University); Malcolm R Macleod (University of Edinburgh); David L Vaux (Walter and Eliza Hall Institute of Medical Research); Eric-Jan Wagenmakers (University of Amsterdam).

We launch this new meta-research section with two important contributions. Iqbal, Ioannidis, and colleagues provide a broad-based evaluation of reproducibility- and transparency-related practices across the biomedical sciences [8]. The authors surveyed a random sample of biomedical articles from PubMed published between 2000 and 2014. They found that the majority of studies did not share raw data, did not provide full protocols, overwhelmingly reported novel findings rather than replications, and did not mention anything about funding or conflicts of interest. Disappointingly, there was little improvement over time, except for the reporting of conflicts of interest. These data quantify the significant shortcomings of current practices and constitute a baseline against which future progress can be evaluated.

Holman, Dirnagl and colleagues use computational modelling and meta-analysis in order to examine the effects of exclusion or loss of animals in preclinical research [9]. In a series of simulations, they find that random loss leads, as expected, to loss of power. However, biased exclusion (e.g., outlier removal) introduces a form of selection bias that dramatically increases the probability of false positives. In a meta-analysis of 100 papers on cancer and stroke, reporting a total of 522 experiments, the authors find that more than half of the studies did not report loss of animals adequately. Importantly, differences in reporting were associated with experimental effect size, suggesting that effect sizes were overestimated.

Readers of the journal will be well aware that these are not the first meta-research articles we have published. In recent years, we have featured several articles in this area in the *PLOS Biology* magazine, many of which would now fit the criteria of our new meta-research section. We have collected these articles here [10], along with key recent meta-research articles published in other PLOS journals.

The *PLOS Biology* magazine will continue to feature meta-research related topics: reporting guidelines, brief surveys, best practices guides, policy perspectives. With our new section on data-driven meta-research, we aim to highlight that research about research is an important area of science. By creating a prominent forum in this field, *PLOS Biology* will contribute to ongoing efforts to improve research standards in the biological sciences and beyond.
