# Correction: Meta-Research: Broadening the Scope of *PLOS Biology*

**DOI:** 10.1371/journal.pbio.1002434

**Published:** 2016-03-30

**Authors:** The PLOS Biology Staff

## Notice of Republication

This article was republished on March 15^th^, 2016 to update the corresponding author’s email address. Please send all correspondence to plosbiology@plos.org and download this article again to view the correct version.
